# Honneth’s dialectical shortcoming: understanding Honneth’s problem with power

**DOI:** 10.3389/fsoc.2025.1686743

**Published:** 2026-01-30

**Authors:** Matthew Rutherford

**Affiliations:** Department of Philosophy, University of Bristol, Bristol, United Kingdom

**Keywords:** ideology, recognition, Allen, Honneth, action-theoretic, system-theoretic, dialectics

## Abstract

Within contemporary critical theory, Axel Honneth’s recognition paradigm continues to exert significant influence. Honneth adopts an empathetically positive view of recognition, that it is always freedom-enhancing. But recently, there is a trend toward a more complex and ambivalent understanding of recognition. Critics highlight Honneth’s inattentiveness to deeper power relations which can use recognition as a tool for domination. This is most evident in the problem of ideological recognition, which is my principal focus. In this paper, I aim to contribute to this literature and offer an explanation for why the problem of ideological recognition arises, and why Honneth struggles to adequately resolve it. I begin by discussing Honneth’s own response to the problem of ideology, which I find wanting. Honneth’s diagnostic criteria are unable to reliably identify and critique ideology. Following this, I then aim to deepen our understanding of the problem of ideological recognition and diagnose a dialectical shortcoming in Honneth’s general theoretical orientation. Namely, in his attempts to pursue an action-theoretic paradigm, Honneth increasingly obscures the mediating role and influence of social systems and their functional logics in shaping spheres of intersubjective action. In failing to adequately attend to this dialectic, Honneth effectively abstracts recognition from its proper social context, and presents a relatively undialectical and idealised vision of intersubjective relations. I conclude by gesturing toward an alternative, a more dialectically informed understanding of social systems and social action.

## 1 Introduction

Given the obvious benefits of recognition and the harms of misrecognition, it is hardly surprising that some—most notably Axel Honneth—adopt an emphatically positive view of recognition; that recognition is always and only a freedom-enhancing endeavour. However, in recent years there is a growing trend toward a more complex and ambivalent understanding of recognition (see Recognition and Ambivalence (Ikäheimo et al., 2021)). Such critics often point to Honneth’s inattentiveness to the subtle ways that recognition can be co-opted and turned into an ideological tool by prevailing power relations. While recognition may be a necessary component of autonomy, it can also be implicated in processes of domination, and hence there is an ambivalence to recognition. This is most clearly evidenced by the problem of ideological recognition, i.e., recognition that serves an ideological and dominating function, which will be my principal focus. I aim to explain why Honneth seems so vulnerable to the problem of ideological recognition. I will attempt to diagnose a shortcoming in Honneth’s general theoretical orientation that helps deepen our understanding of how the problem arises and why Honneth struggles to adequately diagnose ideology. Namely, in his attempts to pursue an action-theoretic framework, Honneth effectively obscures the mediating role of systemic logics in shaping and conditioning spheres of intersubjective recognitive action. To do so, I will first introduce the problem of ideological recognition and Honneth’s recognition-theoretical account of ideology (section 1). Then, I shall find this account wanting (section 2). Finally, I will attempt to provide a more general diagnosis that aims to deepen our understanding of how this problem arises and why Honneth struggles to resolve it (section 3).

## 2 Recognition as ideology

The critique that recognition can be dominating and ideological can be fleshed out in various ways, but perhaps the clearest statement of the problem is provided by Honneth himself (ibid p. 323):

“The act of praising certain characteristics or abilities [i.e., an act of recognition] seems to have become a political instrument whose unspoken function consists in inserting individuals or social groups into existing structures of dominance by encouraging a positive self-image. Far from making a lasting contribution to the conditions of autonomy of the members of our society, social recognition appears merely to serve the creation of attitudes that conform to the dominant system”.

Through the mechanism of social recognition, individuals and/or groups can be lured into occupying or remaining in subordinate social roles. Through the affirmation of certain traits, recognition can foster a self-image in its target that is conducive to prevailing power relations, and it thereby becomes a key mechanism and tool for the reproduction of present patterns of domination. Despite the “presumption of innocence” Honneth granted recognition, it now appears responsible (or at the very least implicated) in the production of ‘happy slaves’; content and fulfilled in their subordination (ibid. p. 325).

Honneth provides some examples. For instance, the sexist ideal of the good mother and housewife (ibid. p. 325–326). Women who strive to fulfil this ideal may receive a degree of social recognition and public esteem and thereby relate positively to this recognitive ideal, but nonetheless they remain trapped within a life of patriarchal domination. Or, to take another example, the recognition and prestige offered to “heroic soldiers” allowed for a “sufficiently large class of men who willingly went to war in pursuit of glory and adventure,” which goes to serves ideological and imperialist ends (ibid. p. 326). In both cases, recognition is implicated and a key mechanism for the creation and fostering of self-conceptions and evaluative attitudes which serve to maintain and reproduce relations of domination. While these examples are obviously ideological and outdated from our “morally advanced present” (ibid. p. 326), it is harder to diagnose ideologies which still have acceptance within present social reality.

Such cases are problematic for Honneth, as they appear (initially at least) indistinguishable from recognition—in fact, they appear *to be* instances of normatively significant recognition. Women who fulfil the ideal of the good housewife, for example, may be able to derive a sense of identity and self-worth, fulfilling their recognitive needs and allowing for the development of positive relations-to-self. All whilst occupying a subordinate social role. As such, it seems recognition is not the unambiguously positive freedom-affirming endeavour that Honneth takes it to be. While recognition may be a necessary pre-condition for autonomy, cases of ideological recognition complicate the picture and gesture toward recognition’s insufficiency.

The task at hand for Honneth is to provide a principled and practical distinction between genuine and ideological recognition in recognition-theoretical terms. To do so, Honneth begins by outlining various conceptual clarification about the nature of recognition. Most importantly, that recognition is not merely symbolic by an “attitude realised in concrete action” (ibid p. 330). To properly recognise another entails material and institutional changes, e.g., to recognise another as a bearer of rights is to treat them accordingly. This is important to emphasise, as it is this aspect of recognition that Honneth ultimately appeals to. Another important clarification to highlight is that recognition does not ascribe qualities to another but rather perceives objectively valuable qualities within them (ibid p. 332–334). This commits Honneth to a value realism where successful recognition correctly responds to objectively valuable traits and qualities. This is an important element of Honneth’s response, which I will address in greater detail in section 2.3.

In addition to these clarifications, Honneth introduces three criteria that ideological forms of recognition must fulfil to achieve their pacifying ideological function (ibid. p. 337–340). To produce ‘happy slaves’, the relevant subjects must accept the recognition offered and to do so they must have seemingly good reasons for doing so. Firstly, the recognition must be *positive*; it must be a positive affirmation with which the subject can positively identify, this excludes obviously discriminatory forms of recognition as they typically harm the recipient (ibid. p. 337–338). Secondly, it must be *contrastive*; it must pick out a distinctive feature as a marker of group identity (ibid. p. 339–340). Thirdly, and perhaps most importantly, the recognition must also be (in the eyes of the recipient) *credible* (ibid. p. 338–339). If it strikes the recipient as incredible and unconvincingly, they will not accept it, and hence it would fail to fulfil its pacifying function. To achieve this, it must use the “evaluative vocabulary of the present” (ibid. p. 338), should it use an outdated and anachronistic evaluative discourse which has long been discredit (as Honneth thinks is the case with the above examples) then subjects would reject it.

For subjects to accept dominating modes of recognition, it must appear to subjects—in its evaluative dimension—to be *prima facie* rational. Should it appear irrational then subjects would likely reject it. Yet Honneth, in line with the traditional understanding of ideology, understands ideology to contain an “irrational kernel” (ibid. p. 328) which the ideology critic aims to reveal. Despite its apparent evaluative rationality, for Honneth ideological recognition nonetheless contains a “second level rationality deficit” (ibid. p. 346); namely, its failure to properly realise itself in material social reality. Ideological recognition contains a gap or contradiction between its evaluative promises and its realisation within social reality (ibid p. 346). In a sense, ideological recognition is all talk and no substance.

Honneth provides the example of the rebranding of the labour-force under neo-liberalism from “wage-workers” to “creative entrepreneurs” (ibid. p. 343). This rebranding uses the evaluative discourse of self-realisation and self-fulfilment. These workers are promised a greater autonomous self-direction in their professional lives. They are not simply labouring but are fulfilling a ‘calling’ or ‘vocation’—or so the ideology goes. This ideological self-conception is conducive to the status quo and thus serves an ideological function. But neo-liberalism is structurally unable to fulfil this promise. In reality, this rebranding accompanies a deregulation of labour and the rise of flexible and precarious work. The evaluative promise of recognition fails to bear fruit in ‘concrete action’, and hence it is ideological and not genuine recognition.

## 3 Problems with Honneth’s account of ideology

Having briefly outlined Honneth’s account of ideology, I now aim to highlight its shortcomings. Particularly by drawing on Allen’s (2010) counterexample of Elizabeth, and from there identify more general limitations and problems in terms of material realisation, evaluative credibility, and Honneth’s value realism.

Elizabeth is a 5-year-old child who has been lovingly raised by her parents (Allen, 2010 p. 25–26). Consequently, she has been able to develop basic self-confidence due to her parent’s recognition. Elizabeth’s parents express and display this recognition (in the form of love and care) through buying her dolls, encouraging her to take ballet lessons, telling her “What a sweet little girl you are” and “how well behaved you are” etc. (ibid. p. 25–26). Even without any conscious intent from her parents, Elizabeth has been recognised and affirmed in accordance with prevailing (and ideological) notions of femininity, which are then internalised and deeply engrained. This is subsequently reinforced through the cultural presentation of femininity in Disney movies and so forth (ibid. p. 26). Elizabeth grows up to be “obsessed” with her looks, prioritises relationships over achievements, is “docile, obedient, and aims to please authority figures” (ibid. p. 26). As Allen puts it, Elizabeth is “receiving recognition (through the vehicle of parental love) and subordinating gender ideology in a single stroke” (ibid. p. 26). Moreover, Elizabeth does not experience this as constraining or demeaning, rather she is “generally content to act out the demands of normative femininity” (ibid. p. 26). As such, this does not produce any recognition struggle, and she is able to successfully derive a sense of identity and self-esteem.^1^

### 3.1 Material realisation

Can Honneth diagnose this case as ideological due to its inability to realise itself in social reality? In this case, however, there seems to be no such gap. As Allen states, “there seems to be no gap at all between the evaluative promise held out by a system of normative femininity and their material fulfilment” (ibid. p. 30). For Allen, those who conform to traditional femininity are better able to navigate the social world and gain “substantial material and economic rewards for doing so, primarily to the marriage of wealthy men,” compared to those who deviate from such norms who continually experience resistance (ibid. p. 30). While I agree with Allen’s general thrust, her reasoning here strikes me as somewhat implausible. Women who do conform to traditional norms still experience resistance, sexism, and abuse—and in many ways this is enabled precisely due to those norms—and not all who conform to such norms marry wealthy men, and indeed those who resist such norms may also marry equally wealthy men.

Despite this, Allen’s point can be made somewhat more directly. The promise of normative femininity is realisable, and indeed has been realised, within present social reality. What Elizabeth was promised, is what she got. What exactly she was promised is a little ambiguous. On the one hand, we may get the impression that it is some level of public esteem for embodying a particular identity and social role, and on the other, it is clear from the example that the recognition Elizabeth receives is love and care, so the promise is the fulfilment of her most basic emotional and material needs. Regardless, both are realisable and are being expressed ideologically. Elizabeth is clearly loved and her needs attended to allowing her to develop basic self-confidence, and she is also able to gain some level of public esteem for her identity and social role—albeit in a more limited sense than men, but in many ways this is a distinct evaluative promise of gender equality, rather than the more basic promise of esteem for a given identity and role.

Furthermore, beyond the case of Elizabeth, there are more general reasons to be sceptical of this appeal to incomplete material realisation. As Wilhelm (2018 p. 137) notes, material changes can accompany ideological recognition. The shift from labourers to creative entrepreneurs follows the deregulation of the labour market. By neo-liberalism’s own evaluative standards, the increase in flexible work options can plausibly be regarded as enabling a greater autonomy and new opportunities for self-realisation, the “standards of judgement here are contestable” (ibid. p. 137). It is therefore more practically difficult to diagnose ideological recognition (from our evaluative present) than Honneth assumes. By its own standards, neo-liberalism can make good on its promises. Ideological shifts in the evaluative spheres, changes in our understanding of autonomy and self-realisation, can follow through to our evaluation of its realisation. The situation is thus more complicated than simply a withheld evaluative promise.

Finally, consider the case of capitalists (such as investment bankers), or state officials (such as border guards and police officers). Clearly both groups can achieve material recognition of their ‘socially valuable labour’, and in the case of capitalists quite significantly so. But nonetheless, they are implicated in the maintenance of problematic social orders and hence are ideological. The ideological content here is not its incomplete realisation, but within its evaluative claim that these are forms of socially valuable labour (or at least more so than other forms of labour). These examples begin to highlight the narrowness and oversimplicity of this criterion which misses the full complexity and diversity of ideology. These cases do not conform to this simple schema of a withheld evaluative promise.

### 3.2 Evaluative credibility

But Honneth’s diagnostic account is not limited solely to material fulfilment. I shall now turn to evaluative credibility. As per Honneth’s account, for ideological recognition to be accepted amongst subjects they must have *prima facie* good reasons for doing so, otherwise they would reject it. However, this response here seems unlikely to work.

Take the criteria that ideological recognition must satisfy to fulfil its pacifying function. The recognition here is positive, it affirms Elizabeth. It is also contrastive; it picks out her femininity as a distinguishing feature and a marker of group identity. Is it credible? Honneth claims that such traditional gender norms [like the ‘good housewife’ (ibid. p. 326)] now strike the recipient as anachronistic and outdated—as they no longer use the “evaluative vocabulary of the present” (ibid. p. 338)—and consequently subjects do not view it as acceptable.

But this simply is not the case. Many women in western societies “quite plainly find the demands of normative femininity to be perfectly credible” (Allen, 2010 p. 30). Allen cites the presentation of women in contemporary popular culture to illustrate this. More recently, we can point to the ‘tradwife’ trend, in which women on social media platforms perform traditional gender roles and present this as a rewarding and desirable lifestyle. Conversely, we can also point to ‘hyper-masculine’ influencers, most infamously Andrew Tate, and their acceptance by young men to illustrate that traditional male gender norms are likewise alive and well. While some find traditional gender norms as outdated and incredible, they quite clearly strike others as being perfectly credible.

Even so, these examples are quite extreme, in the sense that the norms they embody are explicit and perfectly clear. Often, however, such norms weave their way into public discourse and recognitive practices in far subtler ways. Such norms can, and indeed do, make use of in-vogue evaluative discourse. ‘Choice feminism’, for instance, makes use of prevailing evaluative discourse of female empowerment to present regressive and traditional norms as progressive and emancipatory purely as they stem from a women’s choice. The ideological norms at play here are harder to discern and more ubiquitous than their more explicit ‘tradwife’ counterparts. By embracing modern evaluative discourse, such norms can quite easily satisfy the credibility criterion.

While, empirically speaking, it is quite clear that traditional gender norms still have purchase in contemporary society, Honneth can respond that if the relevant subjects knew the true ideological nature of the recognition being offered, they would reject it. Honneth (2009 p. 330) makes such a claim when discussing “normalizing recognition” which is recognition that serves to fix individuals and/or groups to ingrained and normalised identities, roles, and norms.^2^ Honneth argues that such recognition is typically experienced as constraining and as such subjects usually reject it. In cases where subjects do not, we can operate under the assumption that “the concerned parties would actually reject the qualities ascribed to them if they indeed knew all the details” (ibid. p. 339). If Elizabeth knows (or comes to know) that such recognition serves to fix and normalise a certain restricted type of subjectivity, and hence serves an ideological function, she will reject it.

But this assumption is overly optimistic. Elizabeth’s femininity may be a significant source of her sense of identity and self-worth to which she is ‘passionately attached’ (Allen, 2010). Simply because someone has shown her its normalising effect in no way entails that she will reject it. Many individuals acknowledge various gender-based divisions as arbitrary and normalising. Such examples are well-known: pink toys vs. blue toys, men and women’s clothing, the expectation for women to wear make-up and for men not to etc. While some try and subvert such norms, the vast majority thoughtlessly conform and feel their normative weight. Subjects may recognise the arbitrariness of such norms and their normalising function but follow them anyway. The assumption that, should we know all the facts about a normalising or ideological recognition order we would reject it, is simply false.

This problem is further compounded for Allen (2010 p. 26) as for Elizabeth to even contest such norms she must first accept them, and hence risks becoming attached to them. To become a subject, Elizabeth must go through processes of socialisation in which we are recognised by prevailing ideological norms, and only after can Elizabeth rationally reflect on such norms and reject them. But by then, the damage may have been done. However, this can be overstated. Clearly gender norms and roles have evolved, although perhaps less than one would like. So clearly our attachment to subordinating modes of identity can be overcome and changes can occur, even if just to a degree. Moreover, the fact that Elizabeth must accept such norms before she is able to reject them, does not undermine the basis of such critique, but rather attests to its dialectical nature. That it is through the contradictions and limitations of prevailing norms that new norms arise. Allen, in her critique, does not properly appreciate this aspect of Honneth. But nonetheless, and more to the point, Honneth overestimates subjects’ capacity to reject problematic recognitive norms, which continue to hold an implicit subjective credibility in light of their widespread acceptance.

### 3.3 Moderate value realism

While ideological gender norms can, from the perspective of the participants, satisfy the credibility criterion Honneth can reject such recognition by appealing to his ‘moderate value realism’.^3^ In fact, in a later discussion with Allen, this is the response he offers (Honneth et al., 2010). Honneth commits himself to a perceptive model of recognition and a form of value realism. In which we perceive, rather than ascribe, objectively valuable traits and qualities in the other. As such, not any recognition norms will do, but only those that appropriately respond to valuable qualities.

It is easy to see how positing a value realism solves Honneth’s theoretical problem—Elizabeth is being objectively misrecognised—but, as I will argue, it is hard to see how this *practically* helps. How are we to know what such objectively valuable traits and qualities are? After all, ideological norms can masquerade as emancipatory, arming themselves with the “evaluative vocabulary of the present,” as Honneth (2009 p. 327) notes. Indeed, it is precisely because of this that he then appeals to evaluative irrationality and incomplete fulfilment.

For Honneth (2002 p. 508), ‘objective’ values “represent lifeworld certitudes whose character can undergo historical revision,” i.e., a constantly revisable moral consensus, and we come to learn such certitudes through the socialisation processes. In which we “acquire modes of behaviour corresponding to the perception of evaluative qualities,” meaning that we absorb the prevailing valuations and behavioural patterns of present social reality (Honneth, 2009 p. 333). Honneth wants his realism to be open-ended and historically minded. He wants to avoid positing a false universalism that merely generalises the evaluative claims of a specific moment and to continually allow for new developments. There is always the potential for experiences of disrespect to illuminate previously invisible moral blind spots and for increasingly emancipatory interpretations of institutionalised norms (i.e., lifeworld certitudes) to emerge.

This description of how we acquire valuations and what values are (lifeworld certitudes) is empirically plausible, but as yet it lacks the necessary critical edge. Problems of relativism and ideology soon arise as any moral consensus within any given community can become an objective lifeworld certitude. The critical aspect comes with the possibility of contradictions between present practices and reconstructed institutionalised norms. Honneth aims to reconstruct the idealised rational content (i.e., freedom-enhancing content) of lifeworld certitudes, which are brought to the fore via ever-more progressive interpretations. It is on the basis of such expansive interpretations that Honneth critiques prevailing practices as violating their implicit rational content. For instance, the contradiction between modernity’s implicit promise of freedom and its current one-sided interpretation and implementation, which typically elicits experiences of disrespect compelling further moral development. As Honneth states:

“All struggles for recognition … progress through a playing out of the moral dialectic of the universal and the particular: one can always appeal for a particular relative difference by applying a general principle of mutual recognition, which normatively compels an expansion of the existing relations of recognition” (Fraser and Honneth, 2003 p. 152).

The limitations of the status quo seal their own demise through the ‘surplus validity’ of institutionalised principles, to which subjects appeal to in collective moral practices, and which always permit of increasingly inclusive interpretations. So while present lifeworld certitudes suffer from shortcomings, they form part of a progressive “historical learning process” which has a “definite direction” (Honneth, 2009 p. 334). By appealing to progress in our interpretations of lifeworld certitudes, one can discern the direction of moral development, and from this vantage point make “justified judgements about the trans-historical validity of a particular culture of recognition” (ibid. p. 334). Without this notion of progress almost any valuation that obtains socially as an institutionalised norm has a plausible claim to moral validity (ibid. p. 333–334), but by positing an objective direction to history we can diagnose progressions and regressions.

The role of progress within critical theory is a large issue which I cannot fully address here. While progress may be indispensable for left-Hegelian critique, as a defence against ideology it is largely unconvincing, for theoretical, epistemic, and practical reasons. Theoretically, this relationship between normative validity and progress risks granting the new a *prima facie* validity over the old, making it particularly susceptible to new ideological developments. From the presumption of progress, new norms have, all things being equal, a greater claim to validity over old norms. It becomes harder to diagnose new normative developments as problematic, as who is to say that they aren’t moral progressions that we are too anachronistic to accept. New shifts in value-orientations accompanying neo-liberalism have, from the perspective of moral progress, a plausible claim of embodying a progressive historical force. One of the dangers of appealing to progress is that it can imbue new normative developments with a greater validity than they may deserve.

Although, of course, assuming progress does not erase the possibility of regression. While Honneth holds fast to progress, in need not be continuously and naïvely linear. The standard of judgement is not the new-in-itself having preference over the old, but the overall direction of normative development. Nevertheless, epistemically it is still difficult to decipher the direction of historical development. This is especially so given that our historically situated consciousness interprets historical development from the perspective of the present, and thus it can often function as a retrospective justification of the present—as with liberal triumphalist readings of history, e.g., Fukuyama. Also, practically and to reiterate an earlier point, modern evaluative discourse can re-package regressive norms in progressive terminology. Even if we could clearly determine the direction of historical development, ideology may still be practically difficult to identify.

While Honneth is not committed to accepting ideological norms as given, his realism defers to moral norms that obtain socially, and in social contexts of false consciousness and ideology, it remains vulnerable to manipulation—and thus complicating the practical task of diagnosis. While Honneth aims to extract the rational content of norms via increasingly emancipatory interpretations, such norms also contain an ideological content and likewise permit of increasingly ideological interpretations. There is of course no guarantee that these emancipatory interpretations can cement themselves as lifeworld certitudes over ideological interpretations. While new progressive norms may emerge, ideology can frustrate this process, and such ideologies can prove to be stubbornly ingrained, and from the presumption of moral progress, it becomes increasingly difficult to diagnose these new normative developments as problematic.

One can see these various difficulties emerging in Honneth’s direct response to Allen. For Honneth, the recognition offered to Elizabeth is “bad because it is historically superseded” (Honneth et al., 2010 p. 164), it represents a bygone narrowminded phase of our historical learning process. Post feminist interventions, the recognitive norms here can no longer be “publicly and reasonable defended” (ibid. p. 165) and in this way it no longer represents a lifeworld certitude to which we can appeal. Presumably by this Honneth means something like credibly defended in the public sphere, and as these norms are superseded, they fail to withstand public scrutiny as they do not find widespread purchase or support in public evaluative discourse. But again, Honneth is clearly too optimistic, and this recalls the earlier problems Allen identified, these norms still have force in the public sphere. The norms by which Elizabeth is recognised are not particularly shocking but are the kinds of norms widely operative today—hence why it is a compelling example—and in this sense they retain their validity in the public sphere.

Furthermore, the norms by which Elizabeth is recognised are not just ideological. They are both ideological and an enabling condition of her subjectivity which she can plausibly regard—particularly by prevailing evaluative standards—as a condition of her self-realisation. The problem of ideological recognition is not simply that it frustrates the fulfilment of otherwise rational norms or processes of self-realisation, but that it in some way conditions and produces the very selves that subjects seek to realise (Allen, 2010). Hence the problem is more complicated than isolating the rational content of already institutionalised recognitive norms, but that these seemingly rational norms themselves can be implicated in ideological processes. Elizabeth can achieve a positive relation-to-self and a degree of self-realisation, and hence this recognition has a rational content, but she can achieve this on ideological terms. Although, to be clear, this is not to say that Elizabeth’s recognition is unproblematic. While her recognition appears to satisfy Honneth’s normative criteria and slips through his diagnostic net, her recognition is still problematic and ideological as she remains in a subordinate social role. Regardless of how she experiences such recognition, her capacity for flourishing is constrained, as is her ability to contest such recognition (due to her internalisation and acceptance of this recognition)^4^.

## 4 Honneth’s dialectical shortcoming

Thus far I have highlighted the shortcomings of Honneth’s account of ideology. Now I aim to go slightly deeper and explain why this is the case in terms of Honneth’s general theoretical orientation. In pursuing an action-theoretic intersubjective framework, Honneth increasingly obscures the role of social systems in mediating and shaping spheres of intersubjective normative action. In this way, Honneth loses sight of the dialectical relationship of social systems and action. This provides an explanation for why the problem of ideology arises and goes some way in accounting for why Honneth struggles to resolve it.

It is important to note from the outset that such a critique can be overstated. Indeed, one can reconstruct or detect such a dialectical relationship implicitly operating in the background. So Honneth is not simply unaware, nor does he entirely ignore this, but he does fail to appreciate its full importance, and it fails to play a theoretically significant role—Honneth loses sight of this dialectic but does not entirely abandon it. In this way, what is required is less a categorical reformulation, but more of a reorientation or a shift in emphasis. Nonetheless, this shift does move us away from the recognition-paradigm, as intersubjective relations of recognition are no longer the central social-theoretical referent.

To bring these issues to the fore, it will be useful to first recap the motivations that led to the recognition-theoretical turn, which lie in Honneth’s critique and reformulation of historical materialism (Deranty, 2009). Following this exposition, I shall then turn to ideological recognition.

### 4.1 From functional systems to social action

One problem Honneth identifies with Marxism is its economic reductionism; that it implausibly reduces all social spheres to economic activity (see Honneth, 1995a). On this picture, labour operates according to its own immanent logic that is essentially determinant of all other social spheres. In rejecting this hypothesis due to its descriptive and normative implausibility^5^, Honneth seeks an alternative social sphere that is central to social reproduction and adheres to its own immanent logic or ‘grammar’—i.e. intersubjective relations of recognition. In overcoming the labour paradigm, Honneth promotes recognition to be the unifying category of critical theory and the central motor of social development. The central theoretical referent is now struggles over the interpretation of dominant institutionalised moral norms, i.e., struggles for recognition.

The other major sticking point, at least in dominant interpretations of Marx, is its system-theoretic (as opposed to an action-theoretic) orientation. A chief methodological concern for Honneth is to develop a philosophy of praxis that is geared toward practical emancipation (Deranty, 2009 p.13), and only by doing so can one fulfil critical theory’s own premises. System-theoretical approaches emphasise systemic-functional logics^6^ over and above forms of social action, which are understood to be essentially functional expressions of underlying logics. One sees this in Adorno; nothing can withstand the reifying pull of capitalism, which infects everything with its instrumentalist stance. What is of theoretical importance here are systemic logics, the functional working of the whole, and everything else (including social action) is understood only in reference to this logic.

Similarly, structuralist interpretations of Marx (and particularly Althusser) exemplify this for Honneth (Honneth and Joas, 1988 p. 26–31). History is treated as the unfolding of autonomous economic laws which occurs behind the backs of the participants. Subjects become historical “macro-subjects” who are produced by history and are not themselves producers of history (Deranty, 2009 p. 47). Such a perspective clearly is not conducive to an understanding of emancipation through social struggle, as all social action now carries a shade of suspicion of being mere functionaries for the system. But by developing an action-theoretic account, one better facilitates a theory of emancipation.

Not only this, but systems-theories for Honneth fail to develop a convincing theory of social reproduction. For instance, Honneth critiques Horkheimer for failing to follow through on his “communicative” action-theoretic concept of cultural action, which for Honneth would’ve provided Horkheimer with an explanatorily richer social-theoretic account. Honneth (1995b p. 72) states that:

“Only by considering this communicative sphere of everyday social practice could Horkheimer have discovered that societal reproduction never takes place in the form of blind compliance with functional imperatives, but only by way of integration of group specific action norms”.

Horkheimer’s systematic functionalism leads him to effectively eliminate the importance of collective norms of action and (moral) agency in social reproduction. What is missing in this analysis is an action-theoretic perspective and, building on his objections to Marxism’s economism, morally motivated struggles over recognitive norms, which fail to play any major role.

But already here, we can see an early sign of Honneth’s difficulties. In pursuing an action-theoretic model, he risks obscuring the role of systemic logics in conditioning and shaping spheres of intersubjective social action. Collective norms of action are important, there is no denying this, but they are mediated by systemic-functional imperatives and logics. While social integration may occur via “group specific action norms,” these norms themselves are historical productions which are, at least in part, conditioned by the structural contexts in which they operate. The action-theoretic model, in shifting emphasis from functional systems and toward normative action, risks obscuring the ways that systems shape forms of intersubjective action in line with their functional ends.

Although, early on Honneth recognises this danger and acknowledges functional mediations that may condition normative spheres of action. For instance, Honneth (1991 p. 55) bemoans Adorno and Horkheimer for ignoring “the existence of an intermediatory sphere of social action” between functional economic imperatives and subjective ego-formation. While Horkheimer’s notion of cultural action shows initial promise, it ultimately becomes folded into an economic functionalism. In contrast, Honneth positions cultural action as a mediating sphere between economic functionalism and subject-formation, which Adorno and Horkheimer effectively eliminate. So we clearly see Honneth’s sensitivity to this. But he fails to appreciate its full importance, particularly in his account of ideology, and it plays a relatively minor role in his critical theory. The overriding concern with action as a relatively autonomous social sphere has the unfortunate effect of obscuring these mediations which should be at the very centre of the analysis.

This obfuscation also comes clearly into view with Honneth’s turn toward recognition as the central logic of social reproduction. What he risks here is presenting such spheres of intersubjective action with an overly inflated autonomy. In elevating recognition to theoretical primacy, Honneth effectively grants it an independence and autonomy over and above other social spheres. This imbues moral agency and action with an undue (and hence ideological) independence that obscures its systemic mediations. Honneth misses what the likes of Marx, Lukacs, and Adorno saw as essential: understanding how subjective consciousness (and by extension intersubjective relations and norms) are mediated and conditioned by structural logics in line with their functional imperatives.

Thompson (2014) has also observed this. He highlights how Honneth has moved critical theory away from its “core impulse” which is to detail the “ways in which forms of consciousness are shaped and perverted to accept and even legitimize rationalized forms of domination and control” (ibid., p. 781). For Thompson, Honneth commits a neo-idealist fallacy that separates “normative arguments and values from the matrix of social relations shaped by material forms of power” (ibid. p. 780). Honneth abstracts and insulates recognition from wider social structures, but when viewed in their social context, recognition loses its normative purity. Rather, spheres of intersubjective action are “deeply shaped by structural and functional power relations” that rationalise and routinise “the prerogative and goals of social systems” (ibid., p. 782). Consequently recognition can “serve to promote or cement the power relations that already exist” (ibid. p. 780). While Thompson refrains from using the term ideological recognition, this is a clear statement of the problem.

In sum, the priority afforded to spheres of intersubjective social action comes at the expense of social systems and their attendant logics. What is most important for Honneth, social-theoretically, are struggles over the interpretation of institutionalised moral norms. What this misses, or at the very least fails to emphasise, is the role of social systems and their logics (which are not exhausted by their *moral* logics, i.e., its recognition order and moral grammar) in mediating these very spheres of social action and orientating them toward their functional ends. Honneth’s overriding concern with social action tends to obscure this dialectical relationship between systems and action.

### 4.2 Ideological recognition

From here, it is clearer to see how the problem of ideological recognition arises. Social systems can orientate recognition toward their own functional ends. Patterns of recognition cannot freely extricate themselves from systemic logics and structural contexts but are located within them, and as such recognitive norms and practices also feel their influence. Consider again, the case of Elizabeth. The norms by which she is recognised arise via the logics and imperatives of the gender binary and patriarchy, these norms are internalised through the socialisation process (in which recognition is essential) and are then reproduced. The relevant social structures and their functional logics condition and limit the horizon of intersubjective action by imposing a background communicative and interpretative schema. Without doing so, the persistence and pervasiveness of gender norms would remain mysterious.

Thompson (2014) offers a compelling account of this. Systemic logics and imperatives place pressures, “role-expectations,” and obligations upon subjects, shaping their ego-development and value-orientations, which can become internalise and deeply ingrained through the socialisation process and recognition (ibid. p. 783). Like Allen, Thompson focuses on recognition in the family. For Honneth, familial relations of love and care allow subjects to develop basic self-confidence, and healthy recognition relations here are characterised by “primary affectional attachments” (ibid. p. 783). But this characterisation is devoid of the influence of power and the ideologies that permeate in and through the family which co-opt such recognition. As Thompson highlights, “it is through the family that values of consumption, ideas and values about gender, of race, and class and other value-orientations, are introduced and routinized” (ibid. p. 784). In this way, the recognitive sphere of the family can be—and often is—a site for the internalisation of dominant ideologies.

While Honneth may accept this, his action-theoretic perspective does obscure it. Spheres of normative action take centre stage over functional logics and are the primary social-theoretical reference point. In elevating recognition to theoretical primacy, the role of systems fades into the background.

We can also see why Honneth’s recognition-theoretical account of ideology struggles to adequately address the problem. He aims to tackle the problem from within an intersubjective action-theoretic perspective. The focus is on the validity of norms, their subjective acceptance, and their proper implementation. But this focus misses the deeper and more complicated point; that recognitive norms and relations are shaped by systemic logics and the structural contexts in which they operate. Hence such norms can (at least appear) broadly satisfied, and positive relations-to-self can be fostered whilst embodying problematic social norms and occupying subordinate social roles. What is problematic for Honneth is the violation of apparently valid intersubjective recognitive norms, but these norms themselves can have a functional and ideological orientation. Honneth’s intersubjective action-theoretic orientation largely misses this, or at the least struggles to adequately grasp it, as its frame of reference lacks social-theoretical depth. In electing to prioritise spheres of action, Honneth contents himself with recognitive dynamics, but the forces that condition such spheres largely fall by the wayside.

To be sure, however, Honneth can critique functional logics. Should structures systematically cause misrecognition, a recognition-theoretical critique can implicate them in its quest to identify the necessary social conditions for fostering healthy recognition relations—indeed, this forms the normative core of his critique of capitalism. If Honneth can diagnose Elizabeth as being misrecognised, as he thinks he can, then he can diagnose the underlying logics as being pathological in virtue of this. But, as I’ve highlighted, this is a harder task than he assumes, as systemic-functional logics can systematically influence recognitive norms and practices. The norms by which Elizabeth is recognised have a plausible claim to validity (especially given prevailing evaluative standards) and can have a functional content. Such norms can be broadly satisfied and appear to be valid—at least to the point of creating diagnostic difficulties—all whilst cementing ideology. All this serves to complicate Honneth’s otherwise simplistic picture of ideology; it is not simply a failure to realise valid recognitive norms, but that (seemingly) valid recognitive norms can be implicated in the reproduction of ideology. It is this “gravitational pull of functionalist logics”^7^ (Thompson, 2014 p. 782) that Honneth’s action-theoretic orientation struggles to bring into critique.

It is also worth noting that the reproach that Honneth ignores material structures is somewhat misplaced. In fact, Honneth does consider how material factors shape recognitive norms. For instance, Honneth’s own example of ideological recognition, creative entrepreneurs, arises due to neo-liberal deregulation of labour. So clearly Honneth does acknowledge the effects of structures on recognition relations and norms. And in fairness here, Honneth’s account seems better suited to address these examples than that of Elizabeth, as neo-liberalism fails to recognise individuals’ *material* needs (although as Wilhelm highlighted, there may be some difficulties here).

But there are two salient responses here. Firstly, Honneth still maintains that he can neatly distinguish between emancipatory and ideological recognition, and that these are quite clearly ideological. But the preceding discussion gives us reason to be more hesitant. The line between them is far ‘fuzzier’. What is problematic for Honneth is primarily the violation of valid intersubjective moral norms, but (seemingly) valid norms can have a functional orientation. Hence, they can largely be satisfied and cement ideology—as with Elizabeth. While Honneth does acknowledge that structures can affect recognition, it does not really become problematic for him until it adversely affects the quality of recognition, and as the problem of ideological recognition seems to indicate, this is insufficient.

Secondly, the structures that Honneth is most concerned with are, primarily, *normative/moral* structures. This is most clearly evidenced by his preoccupation with the recognition order of modernity [see Fraser and Honneth (2003)] and the “moral economism” of *Freedom’s Right* (Honneth, 2014). This is not to say that Honneth thinks that structures are normative all the way down—he clearly does not think, for example, that economics can be replaced with moral economism—but that this implicit normative logic provides an immanent anchor for critique. But in attending almost exclusively to this moral logic, the mediations and entanglements of moral and non-moral dimensions are left surprisingly under-theorised. They find no real systematic expression in his work, and this again is a consequence of his action-theoretic perspective.

It would also be wrong to accuse Honneth of wholly abandoning dialectical reason. After all, his project is predicated on the left-Hegelian inheritance of a “dialectic of immanence and transcendence” (Fraser and Honneth, 2003 p. 238), and much of his account depends upon the intersubjective recognitive dialectic of self-other. More importantly here is the possibility of dialectical contradictions, and here the critique can be overstated. The norms by which Elizabeth is recognised may play a functional role determined by underlying logics, but there is nonetheless the possibility of contradiction between norms and the structures from which they arise. Systems may produce norms which they cannot satisfy, eliciting experiences of disrespect which galvanises struggle and hence seal their own overcoming—this is perhaps the most important dialectical insight for a critical theory of society that must proceed immanently. While norms may carry ideological and oppressive potential, they also contain a surplus validity that can outstrip present practices.

This dialectical relationship is vitally important for Honneth’s critical theory which he clearly takes as having transformation potential, even if only reformist and institutional [see Schaub (2015) and Honneth (2015)]. If recognition is completely independent of social structures, then it is clearly an ideological abstraction that has no actual effect on the world, which Honneth does not take it to be. Moreover, recognitive norms for Honneth do not appear out of thin air, but they emerge from our social practices and are consequently dialectically intertwined with them.

In Thompson’s critique, he fails to take the possibility of contradiction into proper consideration, and in his argument recognitive norms essentially become little more than mere functional appendages. Hence there is a strand of Thompson’s argument that lapses into an almost totalising functionalism.^8^ But if intersubjective norms are so thoroughly rationalised and ideological, then we move toward a totalising brand of critique which, as Honneth has convincingly shown, is an emancipatory dead-end. We are left with little more than a kind of abstract negation of the social totality, unable to point to social actors and norms of action capable of enacting social transformation and entirely abandoning an action-theoretic perspective (which still retains its importance for these reasons). For my part, the argument presented thus far also suggests something along these lines, but we need not go this far and accept the possibility of contradictions. Recognitive norms can outstrip their structural contexts of application and possess a surplus validity. This grants recognition significant critical purchase which Thompson generally overlooks.

But nonetheless, Honneth’s problems do persist. While contradictions may emerge, ideology can—and does—frustrate this disclosure. There remains the possibility of contradictions coming clearly into view, but also the possibility of ideology smoothing them over, and the action-theoretic perspective struggles to grasp this. Given the practical diagnostic difficulties he encounters—which stem from a dialectic of systems and action—he seems unlikely to effectively illuminate such contradictions. Honneth is too optimistic on the availability of emancipatory norms and their uptake and potency over regressive norms. While we should be wary of overstating the determining influence of systemic logics, and hence lapsing into a totalising critique, the point still stands. In contrast, by admitting a functionalist perspective into the analysis, ideology can come into view more clearly, as it can be (at least partly) understood functionally. Rather than attempting to diagnose ideology from an action-theoretic view, turning to the functional role of recognition may provide more reliable diagnostic guidance.^9^ By emphasising this dialectical relationship between systems and action, Honneth could better allow for an increased social-theoretical depth and overcome the relative surface-levelness of the recognition paradigm.

## 5 Conclusion

In this article, I have offered an explanation for why Honneth struggles to adequately diagnose ideology—due to his action-theoretic perspective he obscures and loses sight of a dialectic of systems and action. This certainly does not undermine the important of recognition for critical social research—it remains an essential element of social life that should be at the heart of any social analysis. Instead, I have tried to highlight its diagnostic limitations and what Honneth misses in turning so decisively toward social action. Which recognition norms are we to pursue, and which are ideological, are hard questions to answer from within an action-theoretic perspective. By—I suggest—emphasising the functionalist dimensions and mediations of recognition relations, it provides a broader social-theoretical framework which can more accurately track recognitive dynamics and their ideological content.

## Footnotes

1 As a reviewer highlighted, this example is largely devoid of countervailing influence. But Elizabeth is a 10 year-old child. Early in her socialisation, she may (plausibly) remain largely under the influence of her primary-caregivers and relatively sheltered from the force of countervailing influences. Children are especially dependent on recognition from their primary-caregivers. As she grows, she will inevitably encounter alternative norms, but by then these norms may be deeply internalised.

2 Normalising and ideological recognition are distinct [see Wilhelm (2018 p.135–6)]. The former ascribes identities to subjects and the latter produces self-conceptions conducive to the status quo. While the two can co-occur, they also may not. But both are ultimately ideological (albeit in different ways) and Honneth’s account aims to critique both. As such, I will treat the two together.

3 I cannot fully address Honneth’s realism here. Instead, I shall limit myself to some remarks on it as a defence against ideology (particularly as outlined in *Recognition as Ideology*).

4 Thus far, I have normatively relied on the suggestive power of the Elizabeth’s recognition and have not articulated an alternative normative criterion. Unfortunately, due to space constraints, I cannot develop this here. But to be clear, Elizabeth’s recognition is problematic as she is constrained in her capacity for flourishing (due to patriarchy and her subordinate social role).

5 For a good overview, see Deranty (2013 p. 749–751).

6 For clarity, the terms ‘systemic-functional logic’, ‘systemic logic’, ‘functional logic’, and ‘structural logic’ are being used here synonymously.

7 This metaphor aims to highlight the organising and structuring capabilities of functionalist logics; they can organise and structure our social world (including intersubjective relations, practices, and norms) to suit their functional ends. For example, complex webs of social interaction (including relevant norms) are all organised toward the accumulation of capital.

8 Thompson thinks he avoids this charge, but unsuccessfully in my view. Due to space constraints, I will not properly defend this claim here. At any rate, Thompson is not my main argumentative concern, but a foil against Honneth.

9 It goes beyond this article to develop this here, but I do want to at least gesture toward a promising direction.
